# Transmigration and spontaneous passage of a gossypiboma documented on contrast study

**DOI:** 10.1016/j.amsu.2018.10.022

**Published:** 2018-10-22

**Authors:** Usman Ismat Butt, Abu Baker Shafiq, Muhammad Umar, Maryam Ashfaq, Mahmood Ayyaz

**Affiliations:** Surgical Unit 2, Services Hospital, Lahore, Pakistan

## Abstract

Gossypiboma or textiloma is a rare but very unfortunate complication of surgery. It refers to a retained foreign body usually forgotten within the abdominal cavity at the end of an operation. It may be a surgical sponge, gauze pad or other form of textile.

We present the case of a middle aged lady who following cholecystectomy had a forgotten gauze which underwent transmural migration and was later expelled via the rectum demonstrated by radiological studies.

## Highlights

• Gossypiboma.
• Transvisceral migration.
• Spontaneous expulsion.
• Radiological study.

## 1 Introduction

Gossypiboma or textiloma is a rare but very unfortunate complication of surgery. It refers to a retained foreign body usually forgotten within the abdominal cavity at the end of an operation. It may be a surgical sponge, gauze pad or other form of textile [1]. Such retained objects act as nidus of inflammation and infection often lead to severe consequences for both the patient and the surgeon including risk of medicolegal consequences and increased morbidity and mortality of patient [2]. Although the exact incidence is unknown this is known to occur mostly during abdominal surgeries [3,4]. The WHO Checklist includes a definite instrument and sponge count at the end of the operative procedure and may help to reduce such complications.

Gossypiboma may be symptom free or it may present with acute or subacute symptoms. The symptoms may include obstruction, peritonitis, adhesions, fistula, abscess formations, erosion into the gastrointestinal tract or extrusion via the rectum. Transmural migration and extrusion is a rare phenomenon.

We present the case of a middle aged lady who following cholecystectomy had a forgotten gauze which underwent transmural migration and was later expelled via the rectum demonstrated by radiological studies.

## 2 Case report

A middle aged lady was in normal health when she was diagnosed with gall bladder stones due to complain of pain right hypo-chondrium and intolerance to fatty diet. She was advised and underwent laparoscopic cholecystectomy at a hospital 6 months ago. Post operatively patient had a smooth recovery however she developed vague abdominal pain in the months following the operation. This was followed by an episode of intestinal obstruction for which the patient was admitted in our hospital. Initially she was managed conservatively while her workup was being done. She was made nil per oral and started on intravenous fluid and electrolyte replacement. A nasogastric tube was passed to decompress and provide rest to the intestines. Mean-while her X-rays were done which however could not pick up the gauze as there were no radio-opaque markers in it. A ultrasound abdomen showed no pathology. She was advised gastrograffin study to determine any narrowing or stricture formation, since she had history of tuberculosis contact. Her study showed a forgotten gauze which by this time had *trans*-migrated into the gut. As serial x-rays were taken the gauze gradually moved along the length of the gut and was eventually passed per rectum by the patient.

After passing out the gauze, the symptoms of the patient resolved. She was allowed orally and kept under observation for 24 hours before being discharged. Patient is currently on follow-up and is symptom free 3 months after this event.

## 3 Discussion

A forgotten foreign body can have disastrous consequences [5]. Although gauzes are chemically inert they cannot undergo decomposition. The body's reaction leads to fibrous deposition and encapsulation. Although it may happen in any surgery, commonly seen in surgeries involving abdomen and pelvis [6]. In the abdomen, gut and omentum may also encapsulate the sponge leading to pressure necrosis and resultant migration partial or complete of the sponge into the lumen. This may lead to fistula or obstruction and the patient may present with symptoms due to them [7]. Exact incidence of gossypiboma is unknown and transmural migration is rarer. Incidence has been reported ranging from 1 case per 5000 to 1 case per 18,000 [8].

**Fig. 1:**
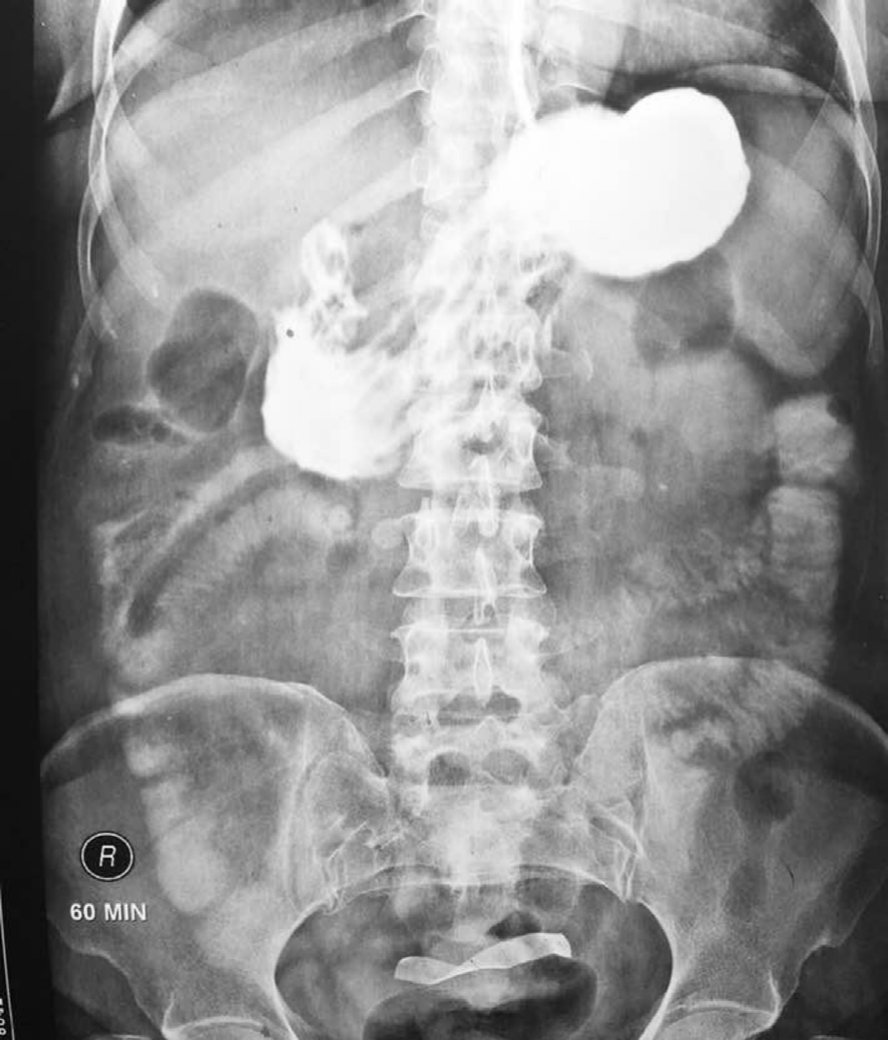
X-ray at 1 hour showing gauze in terminal small gut.

**Fig. 2:**
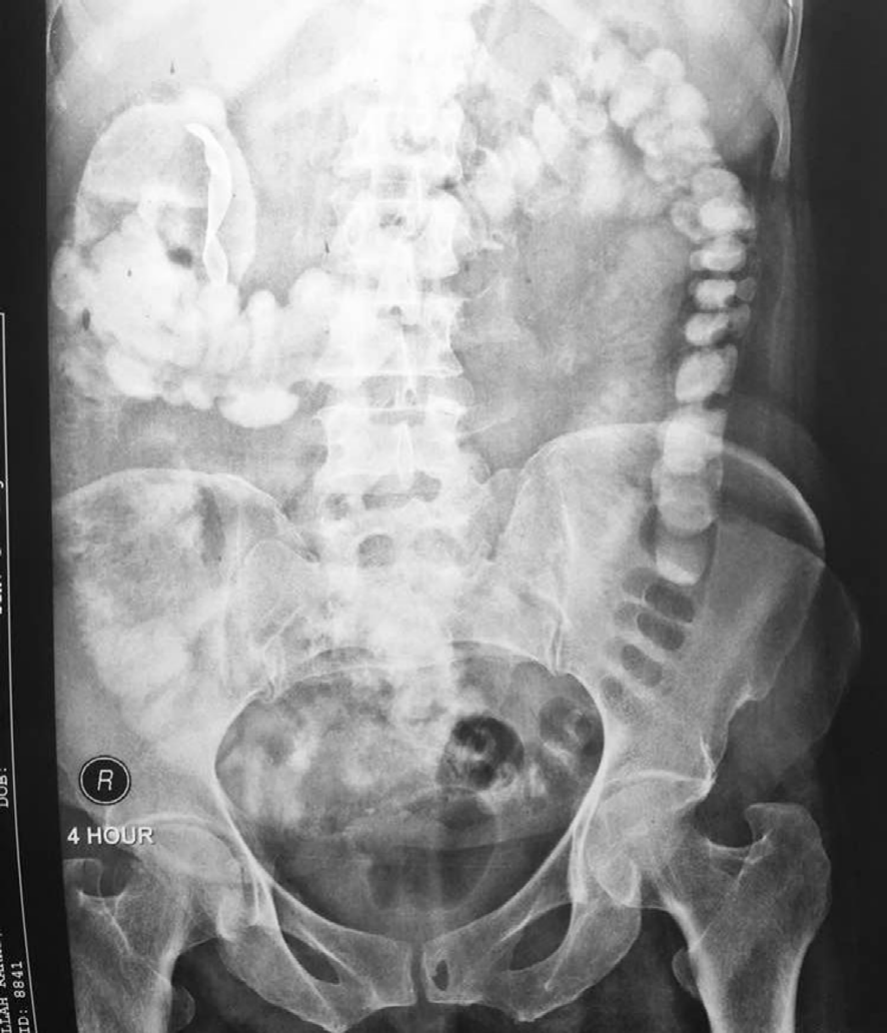
X-ray at 4 hour showing gauze in ascending colon.

**Fig. 3:**
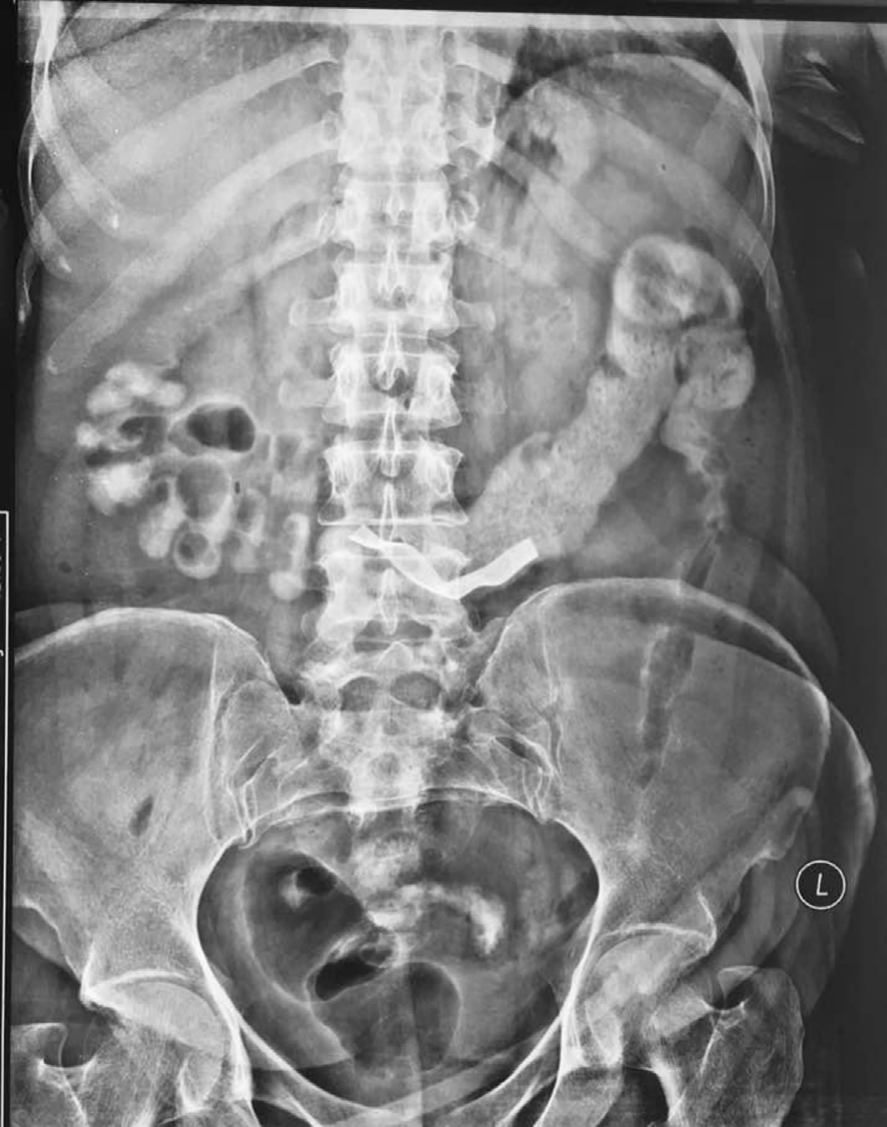
X-ray at 5 hour showing gauze in transverse colon.

**Fig. 4:**
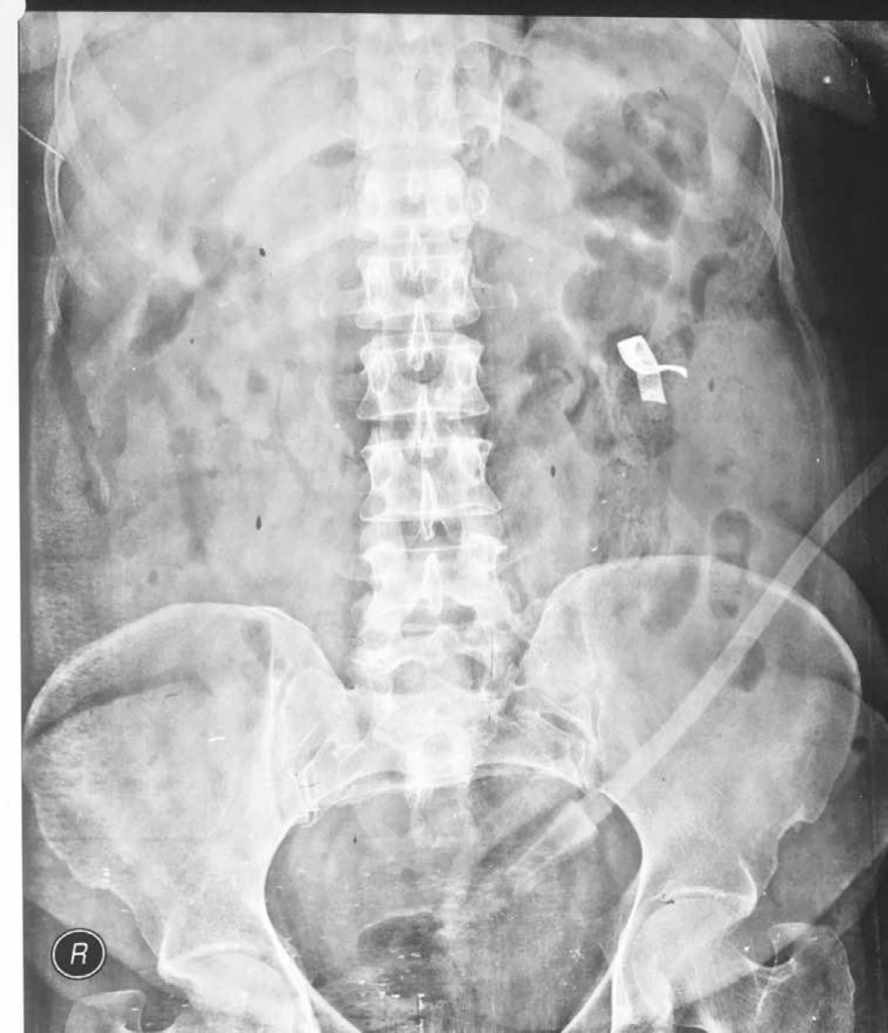
X-ray at 6 hour showing gauze in descedning colon.

When radiopaque markers are used, the sponges may be picked up easily. Often the sponges used in our hospitals lack radio-opaque markers. Gas trapped within sponges may sometimes give it a whirling appearance, however this is often not present. On ultrasound examination they may be picked up as a mass which produces a sharp acoustic shadow, often with wavy internal echoes. CT scan shows a well circumscribed spongiform mass with gas or calcification within the mass giving it a typical un-mistakable appearance [[9], [10], [11]]. Gastrointestinal contrast series may show a well outlined mass often presenting as defect or radio opaque on plain X-rays later on due to retained contrast [12].(see Fig. 1, Fig. 2, Fig. 3, Fig. 4).

Emergency Surgery, unplanned change in operation and BMI of the patients have been identified as clinically significant factors which are associated with retained foreign bodies, with emergency surgery being associated with a 9 fold increase [8]. In 88% of the cases having retained foreign body the sponge count at the end of the procedure was falsely called. Sensitivity of counting was calculated to be only 77% in study by Egorova [13].

Radiologically detectable sponges, avoiding small gauzes in large cavities and methodical examination before wound closure should be done to avoid this dreaded but avoidable complication. Technological advances are now being studied to prevent human error. Radio frequency tagging, barcoding sponges and radio wave detection of impregnated specialized magnetic metal are some of the recent innovations under consideration [[14], [15], [16]].

A number of case reports have been published regarding retained gauzes. There are reports of spontaneous transmigration of gossypiboma into the intestinal tract mostly requiring intervention [[17], [18], [19], [20]]. However very few have documented spontaneous passage of gauze with resolution of symptoms. To the best of our knowledge this is the first reported case where gauze movement was seen during the radiological study itself with expulsion of the gauze soon after the completion of the study and complete resolution of the symptoms of the patient.

This case report has been reported in line with the SCARE criteria [21].

## 4 Conclusion

Gossypiboma is a dreadful but avoidable complication of surgery. It can remain dormant for quite some time before manifesting itself with troublesome symptoms. Adherence to proper count and adoption of new techniques may help to minimize this.

## Conflicts of interest

None.

## Funding

None.

## Ethical approval

Case report.

NO intervention was done. Hence exempt from ethical approval.

## Author contribution

Butt UI involved in data analysis, writing and literature review.

Ahmed AS imvoled in data collection and literature review.

Warraich MU involved in data analysis and literature review.

Ashfaq M involved in data collection and picture collection.

Ayyaz M involved in supervision and guidance during all steps as well as guarantor of final article.

## Registration of research studies

Case report.

## Guarantor

MA will be guarantor of study.

## Disclosure

Nothing to disclose.

## Patient consent

Consent taken from patient.

## Appendix A Supplementary data

SCARE Guideline Checklist tvm.docx SCARE Guideline Checklist tvm.docx
